# How the DNA damage response determines the fate of HTLV-1 Tax-expressing cells

**DOI:** 10.1186/1742-4690-9-2

**Published:** 2012-01-05

**Authors:** Mathieu Boxus, Luc Willems

**Affiliations:** 1 National Fund for Scientific Research, Gembloux Agro-Bio Tech and Interdisciplinary Cluster for Applied Genoproteomics (GIGA), University of Liège, Belgium

## Abstract

How the Human T lymphotropic virus type 1 (HTLV-1) Tax protein stimulates proliferation while triggering cell cycle arrest and senescence remains puzzling. There is also a debate about the ability of Tax to activate or inhibit the DNA damage response. Here, we comment on these different activities and propose a conceptual rationale for the apparently conflicting observations.

## Background

Most cells undergo senescence or apoptosis when they overexpress oncogenes such as Ras, Mos, or Myc *in vitro *[1,2]. First considered as an artifact due to non-physiological experimental settings, oncogene-induced senescence (OIS) has been reproducibly demonstrated to occur in human preneoplastic lesions. It is now accepted that these barriers are triggered in response to unscheduled cellular division and act as innate tumor-suppressive mechanisms *in vivo *[1,2].

Like other oncogenes, the HTLV-1 Tax protein transiently brakes cell cycle progression or even initiates apoptosis and senescence programs [3-13]. On the other hand, Tax also favors cell proliferation by modulating G1/S transition and DNA replication processes [14-16]. *In vivo *cell dynamic studies based on deuterated glucose incorporation methods revealed that HTLV-1 persists because Tax, likely in concert with other viral proteins, actively increases infected cell division [17]. Similar conclusions were drawn from animal models [18-20] supporting the ability of Tax to promote cell division. What dictates the choice between Tax-stimulated proliferation and transient or irreversible cell cycle arrest is still unclear.

### Tax-induced DNA damage and cell cycle arrest

Cell cycle arrest triggered by oncogenes results from an initial highly proliferative state associated with altered DNA replication (e.g. unscheduled firing replication of origins, re-replication or fork collapses) and mitochondrial dysfunctions (increased reactive oxygen species (ROS) production) leading to the formation of double strand breaks (DSB). Subsequent engagement of the DNA damage response pathway (DDR) then halts cell cycle progression [21-23]. If DNA lesions are quickly and properly fixed, cells resume normal proliferation while severe DNA damage drives cells into senescence. Apoptosis is an additional outcome of this process probably depending on the cell type and the extent, the duration or the nature of the damage [21-23].

Overall, onset of DNA damage that alerts the DDR machinery is the critical step in oncogene-induced cell cycle arrest. Recently, we and others described two novel mechanisms by which Tax compromises genome stability [8,16,24]. Tax indeed generates replication-dependent and oxidative DSB, respectively, by modulating the timing of replication origins activation and induction of ROS [8,16]. These Tax-associated activities correlate with activation of several DDR pathway components (ATM, CHK2, H2AX, p53) providing a molecular basis for Tax-induced transient cell cycle arrest, senescence or apoptosis [8,10,16,25,26]. Conceptually, Tax-mediated cell cycle arrest thus appears as a classical DDR whose outcome would be determined by the extent of DNA lesions.

In human cells, DDR signaling cascades converge to trigger two powerful tumor suppressor pathways, p53/p19^ARF ^and Rb/p16^INK4a ^[1]. At a first glance, the fact that Tax evokes senescence independently of these two pathways seems to contradict the DSB/DDR concept [7]. It should, however, be stressed that molecular mechanisms initiating Tax-induced senescence have only been examined in p53-deficient cells [3,4,7]. In this type of cells, Tax-induced senescence (TIS) has been correlated with activation of APC/C and NF-κB, a surge of p21^WAF1/CIP1^/p27^KIP1 ^expression and loss of Skp2 E3-ubiquitin ligase [3-7]. Skp2 inactivation has been proposed as an alternative pathway of cellular senescence in conditions in which the classical p53 and pRb axes are compromised [27,28]. If senescence has a role in HTLV-1 biology, molecular cascades controlling this phenomenon thus remain to be clarified in an appropriate cellular model. On the other hand, Tax-expressing cells can also undergo apoptosis instead of senescence [12,13]. The mechanisms that direct the choice between apoptosis and senescence are still unknown, but likely depend on cellular (e.g. activation status) and viral parameters (e.g. expression levels).

Although *in vitro *HTLV-1 infection halts cells in the G1 phase of the cell cycle [6], experimental evidence of Tax-induced cell cycle arrest and senescence in peripheral blood lymphocytes are indeed still lacking. In the rabbit model the *tax/rex, env and gag/pol *mRNAs were expressed first and at the highest levels immediately after infection [29]. *Ex vivo *culture of primary cells from infected patients also demonstrates that the *tax/rex *mRNA precedes expression of other viral transcripts [30]. Assuming that physiological levels of Tax would be sufficient to arrest cell cycle progression *in vivo*, this process would in fact preclude viral dissemination in the early steps of infection. It is well understood that this phenomenon does not happen. As indicated above, senescence, and more broadly proliferative arrest, occurs in response to a mitotic burst engaged by oncogenic stimuli, a highly proliferative state reproducibly preceding DNA damage formation and DDR stimulation by oncogenes [1,21,31]. Importantly, acquisition of a senescence-like phenotype requires several days of Tax expression [3-8,12,13], while Tax-enforced cellular proliferation is an earlier event [16]. At the molecular level, accumulation of DNA lesions, activation of DDR pathway components and acquisition of senescence markers in Tax-expressing cells occur following S phase acceleration/progression and require at least one passage through mitosis [4,16]. This is an essential observation demonstrating that Tax promotes cell division before triggering senescence. Cell cycle arrest observed in Tax-expressing cells thus appears to be a "collateral damage" originating from uncontrolled proliferation and subsequent genomic instability. In the course of HTLV-1 infection, the DDR pathway could thus act as a failsafe mechanism to remove potentially harmful cells from the proliferative pool. It should be mentioned that Tax expression would also expose cells to a strong immune response [17,32]. In fact, the key question is whether Tax-induced accelerated proliferation systematically ends up in cell cycle arrest and senescence.

### Dual interplay between Tax and the DNA damage response

According to this model, the DDR machinery is a major barrier against Tax-induced proliferation apparently conflicting with a series of reports describing the ability of Tax to inhibit the DDR pathway [25,26,33-35]. These observations are in fact not contradictory and rely on the experimental settings. In the presence of DNA damaging agents, Tax indeed attenuates DDR signaling through sequestration and/or inhibition of critical proteins such as ATM, DNA-PK, CHK1-2 and p53 thereby restricting appropriate response to DNA damage and cell cycle arrest [25,26,33-36]. In the absence of genotoxic stress, expression of Tax is sufficient to trigger strand breaks that alert the DDR machinery. However, Tax would limit the DNA damage response induced by its own genotoxic activities. This model could explain why Tax-expressing cells are still able to reach mitosis and divide despite the presence of activated checkpoints [16]. In the context of infected cells, other viral factors such as HBZ could also contribute by inhibiting senescence induced by Tax [3]. This type of bypass of the DDR barrier has in fact been frequently observed during tumor development [21]. Evasion from senescence and cycle arrest would then allow DNA damaged cells to proliferate, fix and accumulate DNA lesions. While acquisition of lethal mutations would negatively affect infected cell fate, the fixation of growth promoting mutations could pave the way for leukemogenesis.

**Figure 1:**
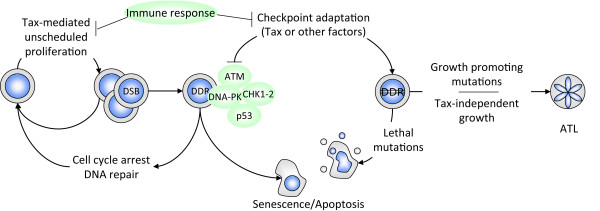
**The interplay between Tax and the DDR pathway dictates cell fate**. In the early steps of HTLV-1 infection, Tax expression fuels unrestrained cells proliferation leading to DNA damage and engagement of the DDR pathway. Severity of genomic lesions determines the outcome of DDR activation: a transient cell cycle arrest allowing DNA repair and cell survival. If the damage cannot be repaired, cells undergo senescence or apoptosis. An intact DDR barrier would thus protect against malignancy. Checkpoint adaptation permits DNA damaged cells to proliferate, accumulate and fix mutations that can have detrimental, neutral, or positive effects on cell growth. Acquisition of growth-promoting mutations is the seed for ATL development, particularly when mutations abrogate the dependence on continued Tax-expression for growth. In this case, cells would indeed evade both DDR and immune response control.

As depicted in Figure 1, we propose that anti- and pro-proliferative functions of Tax are intimately linked and that the fate of Tax-expressing cells is dictated by a subtle balance between unscheduled proliferation, the extent of damage, the strength of checkpoints, and the effects of fixed mutations.

## Conflict of interest disclosures

The authors declare that they have no competing interests.

## Authors' contributions

MB and LW wrote the manuscript. Both authors read and approved the final manuscript.
